# Prevalence and risk factors of premature coronary artery disease in patients undergoing coronary angiography in Kurdistan, Iraq

**DOI:** 10.1186/s12872-015-0145-7

**Published:** 2015-11-18

**Authors:** Ameen Mosa Mohammad, Hekmat Izzat Jehangeer, Sabri Khalif Shaikhow

**Affiliations:** Division of Cardiology, Department of Medicine, Medical School, Faculty of Medical Sciences, University of Duhok, Duhok, Kurdistan Iraq; Duhok Heart Center, Duhok, Kurdistan Iraq

**Keywords:** Premature coronary artery disease, Cardiovascular risk factors, Coronary angiography, Kurdistan, Iraq

## Abstract

**Background:**

Premature coronary artery disease (PCAD) seems to increase, particularly in developing countries. Given the lack of such studies in the country, this study examines the prevalence, associated cardiovascular risk factors, and coronary angiographic profile of the disease in Iraq.

**Methods:**

Data was collected from a total of 445 adult patients undergoing coronary angiography at Duhok Heart Center, Kurdistan in a period between March and September 2014. Patients were divided into PCAD (male <45 years and female < 55 years) and mature coronary artery disease (MCAD).

**Results:**

The prevalence of the angiographically documented PCAD was 31 %. The PCAD had higher rates of hyperlipidemia (*p* = 0.04), positive family history of coronary artery disease (*p* = 0.002), type A lesions (*p* = 0.02), single vessel disease (*p* = 0.01) and medical treatment (*p* = 0.01) than the MCAD. Logistic regression model indicated that male sex (OR 3.38, C.I 1.96–7.22), smoking (OR 2.08, C.I 1.05–4.12), hypertension (OR 1.58, C.I 1.25–2.03), hyperlipidemia (OR 1.89, C.I 1.17–2.42) and positive family history of coronary artery disease (OR 2.62, C.I 1.38–9.54) were associated with the PCAD. Sensitivity analysis showed highest specificity (94.2 %) and positive predictive value (96.5 %) in patients with coronary stenosis >70 % compared to lesser obstruction.

**Conclusions:**

Premature coronary artery disease is alarming  in the country. Cardiovascular risk factors are clustered among them. But the angiographic profile and therapeutic options of PCAD are close to those reported from previous studies.

## Background

Coronary artery disease (CAD) is a major cause of morbidity and mortality worldwide. CAD is a disease usually found in the old. Nowadays, however, it’s often encountered by young adults. It is estimated that about 4–10 % of individuals with documented CAD are less than 45 years [1, 2].

The PCAD is defined, in various studies, as having an age of onset ranging from 30 to 56 years. Clinical studies have affirmed that patients with PCAD have a different clinical presentation, associated CAD risk factors, and coronary angiographic profile compared with the MCAD [2–4].

Patients with PCAD belong to a particular subgroup that needs much more attention since its impact on individuals, families and the society is devastating. Studies on PCAD in certain countries may vary relevant to the populations studied, and the data on PCAD available in Iraq is scarce. This study was aimed at examining the prevalence, clinical presentations, associated cardiovascular risk factors, coronary angiographic profile, and therapeutic options in the PCAD compared to MCAD.

## Methods

### Patients’ recruitment

**Fig. 1:**
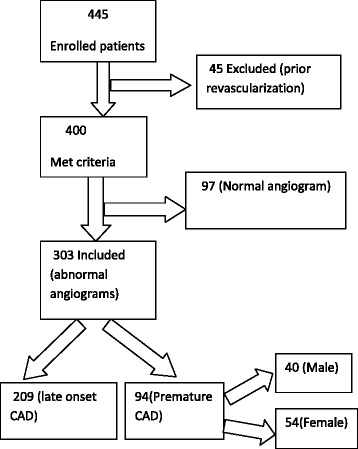
Flow chart of patients’ recruitment. A total of 445 cases were enrolled. 97 cases had normal angiograms and other 45 had prior coronary revascularization and both were excluded

We examined in this cross sectional study a total of 445 clinically diagnosed CAD patients who underwent coronary angiography (CAG) at Duhok Heart Center, Kurdistan, Iraq in the period between March and September 2014. Inclusion criteria included all patients aged ≥18 years, presented with CAD/acute coronary syndrome (ACS), and underwent CAG based on the ACC/ESC indications for CAG for the first time whose coronary angiograms revealed documented coronary lesions [5, 6]. A total of 303 cases (178 men and 125 women) with a mean age 53.8 (SD 5.8) were met these criteria and provided written informed consent. From the recruited sample, 97 cases had normal angiograms and other 45 had prior coronary revascularization (PCI or CABG) and both were excluded from the study (see Fig. 1). CAD manifested in male <45 years and in female < 55 years old was defined a PCAD. Patients were divided into PCAD and MCAD group and the premature group was subdivided into males and females for comparison.

#### Clinical presentations and cardiovascular risk factors

Clinical presentations of patient were classified into chronic stable angina, prior acute coronary syndromes including unstable angina (UA), NSTEMI, and STEMI. Patients were checked for obesity, diabetes mellitus, hypertension, smoking, and family history of coronary artery disease. Diagnoses of these clinical presentations and cardiovascular risk factors were based on international standard definitions [7–14].

#### Coronary angiography

Diagnostic CAG was performed by a team of expert interventional cardiologists. A detailed analysis of angiographic images was done by the operators. Both eye-balling method and quantitative coronary angiography (QCA) were used to estimate the percentage, morphology and length of the coronary lesions. The patients were grouped according the number of major epicardial coronary arteries into one vessel disease (1VD), two vessels disease (2VD), three vessels disease (3VD) or four vessels disease including left main stem (LMS) (4VD). Stenosis of the coronary vessels was considered mild when the luminal diameter was reduced by (<50 %), moderate (50–70 %), and severe >70 % of the original diameter. Complexity of lesions was further categorized according to the joint American College of Cardiology/American Heart Association (ACC/AHA) classification system into: Type A, B and C lesions [15].

#### Statistical analysis

Data analysis was performed by using Microsoft Office Excel 2007and SPSS for Windows, version 16.0. Chicago. Continuous variables were calculated as mean ± (SD), and categorical variables were presented as counts and percentages. A chi-square test was used to compare categorical variables. A student t- test was used for continuous variables. A logistic regression model was used to identify risk factors of premature CAD. *P*-value < 0.05 was regarded as significant. Sensitivity analysis was performed between different categories of coronary artery stenosis percents. The study was approved by the ethical committee at the School of Medicine, Faculty of Medical Sciences, University of Duhok, Kurdistan, Iraq.

## Results

### Comparison between patients with premature and Mature CAD

Prevalence of PCAD is (31 %). Their mean age is 44.2 (SD 5.2). Concerning male sex and STEMI as a clinical presentation, there were a statistical significant difference between the premature and mature CAD (*p* < 0.001, 0.029). There was a significantly higher prevalence rate of diabetes mellitus among MCAD (*p* = 0.002), but a significantly higher rates of hyperlipidemia and positive family history of CAD among PCAD with (*p* = 0.048, 0.002) respectively. Angiographically, there were higher rates of 1 VD, type A lesions and mild coronary stenosis among the PCAD. However, the rates of 3VD and 4VD, type C lesion, and moderate/severe coronary stenosis were significantly higher among the MCAD. CABG seemed to be significantly more common among patients with MCAD (*p* = 0.035). However, medical treatment was more advocated for PCAD (Table 1).

**Table 1:** Comparison between patients with premature and mature CAD

Patient characteristics		Total 303 (100 %)	Premature CAD	Mature CAD	*p*-value
			94(31 %)	209(69 %)	
Mean age ± SD		53.8 + 5.8	44.2 ± 5.2	63.5 ± 6.4	<0.001
Males		178(58.7)	40(42)	138(66)	<0.001
Clinical presentation					
Stable angina		60(19.8.)	23(24.4)	37(17.7)	0.085
UA/or NSTEMI		130(42.9)	41(43.6)	89(42.5)	0.433
STEMI		114(37.6)	30(32)	84(40)	0.029
Risk factors					
Hypertension		140(46)	47(50)	93(44.5)	0.187
Diabetes mellitus		77(25)	14(14.8)	63(30)	0.002
Smoking		81(26)	28(30)	53(25)	0.210
Obesity (BMI > 30 kg/m^2^)		116(38)	38(40)	78(37)	0.303
Hyperlipidemia		69(22)	27(28.7)	42(20)	0.048
FH of PCAD		50(16)	24(25.5)	26(12.5)	0.002
Angiographic characters					
Severity of number of lesions	<50 %	142(21.1)	82(47.4)	60(12)	<0.001
	(50–70 %)	113(16.8)	19(11)	94(18.8)	0.003
	>70 %	418(62.1)	72(41.6)	346(69.2)	<0.001
Type of lesions	Type A	203(30.2)	62(35.8)	130(27.8)	0.024
	Type B	276(41)	71(41.1)	194(41.5)	0.454
	Type C	194(28.8)	40(23.1)	143(30.6)	0.031
Number of major coronary arteries Involved	1VD	109(36)	49(52)	60(28.7)	0.011
	2VD	60(20)	20(21)	40(19)	0.320
	3VD	92(30)	16(17)	76(36)	<0.001
	4VD	42(14)	9(10)	33(16)	0.018
Coronary arteries Involved	LMS	42(6.2)	9(5)	33(6.6)	0.256
	LAD	268(39.8)	74(42)	194(38.8)	0.219
	LCX	170(25.2)	40(23)	130(26)	0.252
	RCA	193(28.6)	50(29)	143(28.6)	0.436
Therapeutic options					
PCI		158(52)	46(49)	112(54)	0.226
CABG		42(14)	8(9)	34(16)	0.035
Medical treatment		103(34)	40(42)	63(30)	0.017

### Comparison between males and females with PCAD

While obesity was significantly more common among females (*p* = 0.038), smoking was obviously more prevalent among males with PCAD (*p* <0.001). There was a significantly higher rate of STEMI among males (*p* = 0.029) as well as the rates of 3VD and 4VD were higher among them (*p* = 0.011 and 0.027) respectively. Therapeutic options showed no significant statistical differences between the two subgroups (Table 2).

**Table 2:** Comparison between males and females with premature CAD

Patient characteristics		Males 40(42.5 %)	Females 54(57.5 %)	*P*-value
Mean age ± SD		40.1 + 4.1	48.3 + 6.3	<0.001
Clinical presentation				
Stable angina		9(22)	14(26)	0.351
UA/or NSTEMI		14(35)	27(50)	0.073
STEMI		17(43)	13(24)	0.029
Risk factors				
Hypertension		20(50)	27(50)	0.500
Diabetes mellitus		5(12.5)	9(17)	0.287
Smoking		24(60)	4(7)	<0.001
Obesity (BMI > 30 kg/m^2^)		12(30)	26(48)	0.038
Hyperlipidemia		11(28)	16(29)	0.410
FH of PCAD		10(25)	14(26)	0.459
Angiographic characters				
Severity of number of lesions	<50 %	40(46.5)	42(48.3)	0.408
	50 %–70 %	8(9.3)	11(12.6)	0.241
	>70 %	38(44.2)	34(39)	0.247
Type of lesions	Type A	26(30.2)	36(41.4)	0.063
	Type B	38(44.2)	33(37.9)	0.201
	Type C	22(25.6)	18(20.7)	0.222
Number of major coronary arteries Involved	1VD	16(40)	33(61)	0.229
	2VD	8(20)	12(22)	0.374
	3VD	10(25)	6(11)	0.011
	4VD	6(15)	3(5)	0.027
Coronary arteries Involved	LMS	6(6.9)	3(3.4)	0.148
	LAD	37(43)	37(42.5)	0.172
	LCX	18(20.9)	22(25.3)	0.364
	RCA	25(29)	25(28.7)	0.100
Therapeutic options				
PCI		19(47.5)	27(50)	0.405
CABG		5(12.5)	3(6)	0.116
Medical treatment		16(40)	24(44)	0.333

### Risk factors of PCAD

Using PCAD as a dependent variable, independent CAD risk factors among PCAD including male gender, arterial hypertension, diabetes mellitus, smoking, hyperlipidemia, and family history of CAD were assessed by logistic regression model. The analysis revealed that male gender, hypertension, smoking, hyperlipidemia, and family history of CAD were independent risk factors for PCAD (Ps < 0.05) (Table 3).

**Table 3:** Logistic regression model of CAD risk factors in premature CAD

CAD risk factors	Odds ratio	Confidence interval	*p-value*
Male gender	3.3807	1.9644 to 7.2263	<0.001
FH of CAD^a^	2.6211	1.3872 to 9.5412	0.0113
Hyperlipidemia	1.8963	1.1770 to 2.4271	0.0249
Smoking	2.0840	1.0520 to 4.1281	0.0353
Hypertension	1.5896	1.2517 to 2.0365	0.0485

^a^FH = Family History

Sensitivity analysis of different categories of coronary artery stenosis showed highest specificity and positive predictive value (PPV) in patients with coronary stenosis >70 % obstruction compared to other categories of lesser obstruction (Table 4).

**Table 4:** Sensitivity analysis of different categories of coronary stenosis

Coronary stenosis (%)	Sensitivity %	Specificity %	PPV^a^ %	NPV^b^ %	Accuracy %
<50 %	96.2	90.7	92.0	94.4	95.2
≥50 %–≤ 70 %	95.1	91.2	93.6	92.7	94.3
>70 %	91.4	94.2	96.5	89.9	93.8

^a^PPV = Positive predictive value. ^b^NPV = Negative predictive value

## Discussion

This study showed an alarming higher rate of PCAD compared to most of reports and studies conducted worldwide. This rate is higher than rates reported by Mohammad et al. from Iraq, Prashanth et al. from Oman and Al-Nozha et al. from Suadia Arabia [16–18]. At the same time it is much higher than the reported rates of documented CAD in young populations in USA, India, China and Japan [19–22]. On the contrary, it is lower only than the rate reported exclusively by Zahrni et al. who found 55 % of CAD in Malaysian is of premature onset, and this is probably related to the mean age difference between both studies [23]. The age of PCAD presentation in the current study is much younger than the mean age for Zahrni et al., and is comparable to Roxona Sadeghi et al. from Iran [23, 24].

In consistence with the results found by Zahrni et al., Tahir et al., and Prashanth et al., the current study showed higher incidence of ACS compared to chronic stable angina among premature group [17, 23, 25]. But Abu Siddique et al. showed higher rates of chronic stable angina and not ACS among PCAD [26]. Interestingly among the latter group, males are more likely to present with STEMI as compared to females. This finding has also been demonstrated in other observational and retrospective analyses worldwide. This group of patients represents an area for further study and possible future interventions. Currently, there are prospective studies ongoing to evaluate subclinical markers of atherosclerosis and indeed some currently unknown risk factors may be responsible for this finding [27, 28].

This study, like Nafakhi though unlike Abu Siddique et al., revealed no significant difference in the prevalence of hypertension between premature and mature CAD [26, 29]. Noeman et al. found similar rate to the current study of hypertension among PCAD in Pakistan [30]. Compared to Prashanth et al. and Mahnoosh Foroughi et al., while this study found a lower rate of obesity among PCAD, it concluded a similar one among female gender [17, 31]. This gender difference could be attributed to many etiologies including cultural customs and social norms of the society.

The study, in accordance with Tahir et al. and Badran et al., showed a significant difference in the prevalence of diabetes mellitus between premature and mature CAD. Unlike Badran et al., however, it showed no significant gender difference of prevalence of diabetes mellitus in the PCAD [25, 32]. Besides, the study showed a significantly higher incidence of dyslipidemia in PCAD that is similar to what Penida et al. found [33].

The rate of smoking among PCAD was lower in this study compared to Roxana Sadeghi et al. (30 % vs. 46 %) [24]. Smoking was rare among females in this study likely because yet it is not popular among women in the Kurdish society. Positive family history of CAD among the premature group of this study is similar to Mahnoosh Foroughi et al. study from Iran [31]. Nevertheless, higher percentages of positive family history were found in Turkey by Yildrim et al. [34]. Though recognized as an important risk factor for ACS in PCAD, HIV significantly impacts the trend of mortality increase in such patients. Interestingly, however, our study did not show any registered cases of which HIV caused PCAD [35].

Similar to Ibrahim Shah et al. and Badran et al. studies we found a significant higher rate of mild coronary lesions among premature group though it is in contrast to S. Sadiq Shah et al., who reported no statistical difference in the severity of coronary lesions between premature and mature CAD [32, 36, 37]. This finding, in the current study, possibly related to short duration of clustering of risk factors among premature group.

Evaluating the types of coronary lesion, Tahir et al. reported no statistical difference between premature and mature CAD. This study showed a significant higher rate of type A lesions among PCAD [25]. In consistence with Badran et al., Almayali and Christus et al., the study found higher rate of single vessel disease among PCAD [32, 38, 39]. Conversely, it showed a significant higher rate of 3VD/ 4VD among mature group. Similar results were reported by many studies, including Tewari et al. [25, 32, 39, 40]. In contrast with Farhan et al., this study showed a significantly higher rate of 3VD and 4VD among males with PCAD [41].

In accordance with the Abu Siddique et al. and Nafakhi, the distribution of major epicardial coronary artery involvement in this study was independent of the age [26, 30]. The therapeutic options, including the CABG that were significantly more doable in the MCAD and medical treatments that were more common in PCAD provided that there was less complex angiographic profile among PCAD compared to MCAD, were similar to Chrustus et al. [39].

### Study limitations

There are a few main limitations to this study. To start with, only patients presented with symptoms and assigned for an angiogram were diagnosed with CAD that might limit the generalizability of the results. Though the study was a single center experience that could result in a referral bias, it reflected the trends of prematurity of CAD all over the country given a close attitude, genetics and socio economic similarities among the population. No data on follow up as well as the coronary imaging (e.g. IVUS) findings of patients included were collected. Nevertheless, this study, which is the first detailed one conducted on PCAD in the country, identified an unmet scientific need in the area, enrolled a relatively large sample size to investigate the prevalence of PCAD, and clearly defined data on all patients.

## Conclusions

This study concludes that the rate of premature CAD in the country is alarming and that is mostly related to the clustering of cardiovascular risk factors. To reduce rates and consequences of PCAD, it is paramount to control the cardiovascular risk factors, screen the susceptible populations at risk and improve the coronary interventional services.

## Abbreviations

ACS: acute coronary disease
CABG: coronary artery bypass grafting
CAD: coronary artery disease
CAG: coronary angiography
EF: ejection fraction
FH: family history
LAD: left anterior descending artery
LCX: left circumflex artery
MCAD: mature coronary artery disease
NPV: negative predictive value
PCAD: premature coronary artery disease
PCI: percutaneous coronary intervention
PPV: positive predictive value
QCA: quantitative coronary angiography
RCA: right coronary artery
VD: vessel disease
